# Inhalation of Atmospheric-Pressure Gas Plasma Attenuates Brain Infarction in Rats With Experimental Ischemic Stroke

**DOI:** 10.3389/fnins.2022.875053

**Published:** 2022-04-19

**Authors:** Ye Chen, Bingyan Yang, Lixin Xu, Zhongfang Shi, Ruoyu Han, Fang Yuan, Jiting Ouyang, Xu Yan, Kostya Ken Ostrikov

**Affiliations:** ^1^Department of Pathophysiology, Beijing Neurosurgical Institute, Beijing Tiantan Hospital, Capital Medical University, Beijing, China; ^2^School of Basic Medical Sciences, Capital Medical University, Beijing, China; ^3^School of Physics, Beijing Institute of Technology, Beijing, China; ^4^School of Chemistry and Physics and Centre for Biomedical Technologies, Queensland University of Technology, Brisbane, QLD, Australia

**Keywords:** atmospheric pressure plasma, plasma medicine, ischemic stroke, magnetic resonance imaging, neuro-protection

## Abstract

Previous studies suggest the potential efficacy of neuroprotective effects of gaseous atmospheric-pressure plasma (APP) treatment on neuronal cells. However, it remains unclear if the neuroprotective properties of the gas plasmas benefit the ischemic stroke treatment, and how to use the plasmas in the *in vivo* ischemic stroke models. Rats were subjected to 90 min middle cerebral artery occlusion (MCAO) to establish the ischemic stroke model and then intermittently inhaled the plasma for 2 min at 60 min MCAO. The regional cerebral blood flow (CBF) was monitored. Animal behavior scoring, magnetic resonance imaging (MRI), 2,3,5-triphenyltetrazolium chloride (TTC) staining, and hematoxylin and eosin (HE) staining were performed to evaluate the therapeutic efficacy of the gas plasma inhalation on MCAO rats. Intermittent gas plasma inhalation by rats with experimental ischemic stroke could improve neurological function, increase regional CBF, and decrease brain infarction. Further MRI tests showed that the gas plasma inhalation could limit the ischemic lesion progression, which was beneficial to improve the outcomes of the MCAO rats. Post-stroke treatment with intermittent gas plasma inhalation could reduce the ischemic lesion progression and decrease cerebral infarction volume, which might provide a new promising strategy for ischemic stroke treatment.

## Introduction

Stroke, the second leading cause of death globally, is a kind of cerebrovascular disorder characterized by the sudden rupture of cerebral vessels or blockage of blood vessels, namely hemorrhagic stroke and ischemic stroke, respectively (Kuriakose and Xiao, 2020). Ischemic stroke as the most common type of stroke accounts for approximately 70% of all incident stroke cases (Wang et al., 2017). It is caused by an abrupt and sustained reduction in regional cerebral blood flow (CBF), leading to irreversible neuronal death in the core of the lesion (Meschia and Brott, 2018). Therefore, clinicians generally perform reperfusion therapies to restore the blood supply and to salvage the penumbra as soon as possible, such as intravenous thrombolysis, mechanical thrombectomy, and surgical bypass (Hwang et al., 2011; Otsu et al., 2020). However, due to irreversible neuronal death and non-renewability of neuronal cells, the prognosis is always poor despite the application of reperfusion therapies. Therefore, appropriate neuroprotective strategies after the occurrence of ischemic stroke could protect the neuronal cells threatened by ischemic damage and delay the ischemic core progressing, which could save more time for the reperfusion therapies and further improve the prognosis (Baron, 2018).

Since the early use of atmospheric-pressure plasma (APP) generated in gases for sterilization in the mid-1990s (Laroussi, 1996), the APP has emerged as a promising technology for medical applications. The plasma features a low gas temperature, abundant reactive oxygen and nitrogen/oxygen species (RNS/ROS), and importantly, no significant genotoxic effects have been reported (Weltmann and Von Woedtke, 2016). These advantages make the APP appealing for clinical trials, while some APP sources were CE-certified as medical devices (Weltmann and Von Woedtke, 2016). Atmospheric-pressure plasma jet (APPJ) is one of the most popular plasma sources due to its open geometry (which means that the plasma could be generated under an open atmospheric environment and suitable for various applications and objects of different sizes or shapes) and easy operation in medical practice and clinical environments. Based on the oxidative and/or nitrative stress produced by the APPJ, it has a wide range of applications in pathogen elimination and cell treatment, including inactivation of a broad spectrum of microorganisms and killing of cancer cells with higher doses (Daeschlein et al., 2014; Khalili et al., 2019; Dai et al., 2020; Chen et al., 2021). Meanwhile, the APPJ treatment could also be “double-edged sword” since RNS and ROS at physiological concentrations are important intracellular signaling molecules involved in many positive physiological benefits (Valko et al., 2007), such as immune responses, cell proliferation, and tissue regeneration with lower doses (Lee et al., 2017; Katiyar et al., 2019; Przekora et al., 2019). In the central nervous system (CNS), a recent study by Tian et al. (2021) showed that plasma treatment induced reactive oxygen and nitrogen species (RONS)-elicited neuroprotection against glutamate excitotoxicity by activating glutathione and erythroid 2-related factor 2 (Nrf2) (Tian et al., 2021). Our previous studies also found that the neuroprotective properties of plasma were regulated by activating nitric oxide (NO)/cyclic guanosine monophosphate (cGMP)/protein kinase G (PKG) pathway and inhibiting mitochondrial apoptosis pathway (Yan et al., 2020, 2022). Another study by Jang et al. (2018) demonstrated that the mechanism of atmospheric plasma inducing neural differentiation was regulated by the RONS productions and the activation of Trk/Ras/ERK signaling pathway, thus indicating potential applications of the plasma in nerve injury treatments.

Our previous studies have demonstrated that APPJ could be an effective neuroprotective agent in the multiple pathological injury processes during ischemic strokes, such as oxidative stress (Yan et al., 2017), hypoxia (Yan et al., 2018), and oxygen and glucose deprivation (OGD) (Yan et al., 2020). However, how to use APPJ for the ischemic stroke treatment is still unknown. The current study aims to evaluate the therapeutic efficacy of APPJ in a rat ischemic stroke model using magnetic resonance imaging (MRI), animal behavior scoring, and 2,3,5-triphenyltetrazolium chloride (TTC) staining.

## Materials and Methods

### Animals

Each animal experiment was carried out in line with the Guidelines for the Care and Use of Laboratory Animals and was approved by the Animal Care and Use Committee at Beijing Neurosurgical Institute (No. 201902030). Fifty-four 8–10-week-old male Sprague-Dawley rats were purchased from the Charles River Experimental Animal Technology, Co., Ltd. (Beijing, China). Rats were raised in the temperature-controlled environment under the light–dark cycle of 12 h/12 h, and they were allowed to drink water and eat food freely. Surgery was performed on the rats when their weights reached 250 ± 10 g. All possible steps were taken to minimize animal sufferings and the number of rats used in the study.

### Plasma Source and Treatment

**FIGURE 1:**
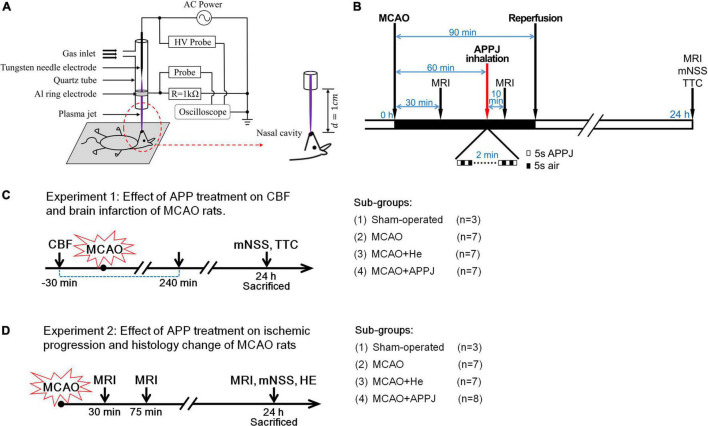
APPJ device and treatment protocol. **(A)** Diagram of the experimental setup. **(B)** Experimental procedure. **(C)** Rats successfully received MCAO surgery were randomly divided into MCAO, MCAO + APPJ, and MCAO + He groups. Then rats in each group have to be further randomly divided into two subgroups for different experiments, rats in one subgroup sequentially received CBF monitor, and sacrificed for TTC staining. **(D)** Rats in another subgroup sequentially received the MRI test, the behavioral test, and sacrificed for HE staining at indicated time points. APPJ, atmospheric-pressure plasma jet; CBF, cerebral blood flow; HE, hematoxylin and eosin; MCAO, middle cerebral artery occlusion; TTC, 2,3,5-triphenyltetrazolium chloride.

The plasma jet was generated by a tungsten needle-aluminum ring structure based on a dielectric barrier discharge (Figure 1A). Details of the plasma source equipment could be found in our previous study (Yan et al., 2020). The applied AC voltage and discharge frequency were fixed at 5.6 kV and 5 kHz, respectively. About 1.4 slm of He (99.999% pure) was used as the feed gas. We utilized a spectrometer (AvaSpec-ULS3648) for monitoring the optical emission spectroscopy (OES) of the plasma jet. Rats were laid flat on a *z*-axis displacement platform for treatment, and we positioned their nasal cavities at 1 cm under the quartz tube nozzle as shown in Figure 1A. The effluent of the plasma jet was sprayed directly into the rat’s nasal cavity for inhalation.

### Middle Cerebral Artery Occlusion and Reperfusion Treatment

Rat ischemic stroke model was conducted through intraluminal occlusion of the right middle cerebral artery (MCA) according to the previous description (Shi et al., 2021). After anesthesia with the intraperitoneal injection with xylazine/ketamine mixture (10 and 100 mg/kg, respectively), each rat was put in a supine position and a cervical incision was made.

The left common carotid artery (LCCA), external carotid artery (ECA), and internal carotid artery (ICA) were exposed with the microscope. The silicon-coated filament (diameter, 0.36 mm; Cat. No. 2636A5, Cinontech Co., Ltd., Beijing, China) was advanced from ECA to ICA till the feeling of resistance (about 18–20 mm), so as to ensure that the MCA origin was occluded. After 90 min of embolization, the filament was slightly retrieved for achieving ischemic-reperfusion. Then, the ECA stump was tightened and the neck was sutured. The identical surgery was performed in sham-operated rats (*n* = 6) with no insertion of a filament. Rats that successfully received middle cerebral artery occlusion (MCAO) surgery were randomly divided into three groups: MCAO, MCAO + APPJ, and MCAO + helium (He). Then rats in each group have to be further randomly divided into two subgroups for different experiments: rats in one subgroup sequentially received CBF monitor and sacrificed for TTC staining; and rats in another subgroup sequentially received the MRI test, the behavioral test, and sacrificed for hematoxylin and eosin (HE) staining (Figures 1C,D).

### Physiological Parameters Monitoring

During the operation, we used a temperature-controlled operating table and heating blanket to maintain the rat core temperature (rectal) at 37°C. The regional CBF (5 mm lateral, 1 mm posterior to bregma) was monitored during the surgery with the laser Doppler flowmeter (PowerLab 8/35, ADInstruments, Colorado Springs, CO, United States). By adopting the low-speed dental drill and the stereotaxic apparatus, we prepared a 2-mm burr hole on the skull. We affixed the needle-shaped laser probe tip onto the rat’s skull surface on the brain region to record the CBF. The finger clip was clamped on the rats’ hind paws to monitor the blood oxygen saturation (SpO_2_), which was recorded by the PowerLab system.

### Experimental Design

Rats with CBF reduced to about 25% of baseline were considered as successful of MCAO and randomized into 3 groups: (1) MCAO group (*n* = 16), (2) MCAO with the APPJ treatment group (*n* = 16), and (3) MCAO with He inhalation group (*n* = 16). At 30 min after the MCAO surgery, rats were examined with MRI to confirm whether the ischemic stroke model is successful. At 60 min, rats received in total 2-min intermittent inhalation of APPJ or He (cycles of 5 s APPJ/He and 5 s air). At 10 min after the treatment, the rats received an MRI examination to evaluate the response to the APPJ treatment. The rats in the MCAO group received no treatment and received MRI examination with the other two groups. At 90 min, the filament was removed for restoring the local CBF to mimic the reperfusion therapies of the ischemic stroke. At 24 h, rats were examined through the behavior scoring and MRI again and were then sacrificed for the HE staining. The experimental design is summarized in Figures 1B–D. Five rats [2 in MCAO, 2 in MCAO + He (gas only), and 1 in MCAO + APPJ groups] died due to subarachnoid hemorrhage, which were excluded from subsequent analyses.

### Magnetic Resonance Imaging Testing

The 7.0-T scanner (BioSpec, Bruker, Billerica, Germany) with a four-channel phased-array rat head coil was used for MRI examinations of rats (*n* = 7 for the MCAO group, *n* = 7 for the MCAO + He group, and *n* = 8 for MCAO + APPJ group). The spin-echo echo-planar-imaging sequence was utilized to obtain diffusion-weighted imaging (DWI) with the following parameters: matrix = 128 × 108, field of view (FOV) of 4 × 4 cm, repetition time (TR) of 4,500 ms, slice thickness (THK) of 1 mm, echo time (TE) of 35 ms, *b*-values of 1,000 s/mm^2^, and number of averages (NA) of 1. Later, 2 different *b*-values (0, 1,000 s/mm^2^) were utilized to calculate the quantitative maps of apparent diffusion coefficient (ADC). The Radiant Viewer^1^ was employed to analyze the images. On ADC maps, we quantified the lesion volumes (mm^3^) according to our previous study (Fang et al., 2016).

### Behavioral Tests

For each rat, a researcher blinded to grouping carried out several behavioral tests at 24 h post-MCAO. As previously detailed (Chen et al., 2001), modified neurological severity score (mNSS), an 18-point ordinal scale, was used to evaluate the severity of nerve injury in MACO rats. mNSS is mainly divided into four parts: motor tests, sensory tests, beam balance tests, and reflexes absent and abnormal movements, which were detailed in Table 1. When the rat could not complete the tasks or did not have a tested reflex, it was rated 1 point; and the higher the score, the more severe the injury.

**TABLE 1:** Neurological severity scores scale.

	Points
Motor tests	
Raising rat by the tail	3
1 Flexion of forelimb	
1 Flexion of hindlimb	
1 Head moved > 10°to vertical axis within 30 s	
Placing rat on the floor (normal = 0; maximum = 3)	3
0 Normal walk	
1 Inability to walk straight	
2 Circling toward the paretic side	
3 Fall down to the paretic side	
Sensory tests	2
1 Placing test (visual and tactile test)	
2 Proprioceptive test (deep sensation, pushing the paw against the table edge to stimulate limb muscles)	
Beam balance tests (normal = 0; maximum = 6)	6
0 Balances with steady posture	
1 Grasps side of beam	
2 Hugs the beam and one limb falls down from the beam	
3 Hugs the beam and two limbs fall down from the beam, or spins on beam (>60 s)	
4 Attempts to balance on the beam but falls off (>40 s)	
5 Attempts to balance on the beam but falls off (>20 s)	
6 Falls off: No attempt to balance or hang on to the beam (>20 s)	
Reflexes absent and abnormal movements	4
1 Pinna reflex (head shake when touching the auditory meatus)	
1 Corneal reflex (eye blink when lightly touching the cornea with cotton)	
1 Startle reflex (motor response to a brief noise from snapping a clipboard paper)	
1 Seizures, myoclonus, myodystony	
Maximum points	18

### Brain Infarction Size Determination

Each rat was given euthanasia at 24 h post-MCAO. The brain was immediately dissected and frozen, then the coronal sections (2 mm) of the brain tissues were prepared and stained with the 2% 2,3,5-triphenyltetrazolium chloride (TTC) (Solarbio, Beijing, China) for 30 min in the dark and fixed in formalin to visualize the infarction. The infarcted and healthy tissues were stained white and red, respectively. After the photography, we adopted the Image-Pro Plus 6.0 for calculating the areas of the contralateral hemisphere (C_i_), ipsilateral hemisphere (I_i_), and ipsilateral non-ischemic region (N_i_) in each section (i). The percentage of infarct volume was calculated and corrected for edema using the following formula according to a previous study (McBride et al., 2015):


$$\mathrm{Infarct}\mathrm{volume}\left(\%\right)=100\times \frac{\sum_{\text{i}}\left(\left(\frac{{\text{I}}_{\text{i}}-{\text{N}}_{\text{i}}}{{\text{I}}_{\text{i}}}\right)\,{\text{C}}_{\text{i}}\right)}{2\,\sum_{\text{i}}{\text{C}}_{\text{i}}}$$

### Histology Examination

The HE staining was performed for histology examination. Brain tissues in each group were collected and fixed 10% paraformaldehyde. The tissue sections were dehydrated and processed in the automatic tissue processor (ASP300S, Leica Biosystems, Buffalo Grove, IL, United States Leica) and were immersed in paraffin. A 4-μm paraffin section was then prepared. HE staining was performed after dewaxing and hydration. The slides were scanned with the Leica Aperio AT2 scanner (Leica Biosystems, Buffalo Grove, IL, United States).

### Statistical Analyses

The data were presented in a form of mean ± SEM and analyzed by the Prism software package (version 8.2.1). The Brown–Forsythe test was used to test the normality. One-way ANOVA tests were used when data were normally distributed, and Tukey’s multiple comparisons were adopted to compare between multiple groups. The Kruskal–Wallis one-way ANOVA test was applied in the case of non-normal distribution. In each of the statistical tests, *p* < 0.05 was considered as statistical significance.

## Results

### Plasma Produces Mild and Effective Treatment Conditions

**FIGURE 2:**
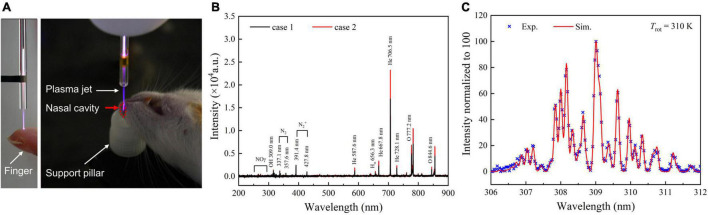
Plasma process and characteristics ensure mild and effective treatment conditions in animal models. **(A)** Images of the plasma inhalation treatment of rats with experimental ischemic stroke. **(B)** OES of the plasma jet at the quartz tube nozzle when APPJ is ejected into the air (case 1) or into a rat’s nasal cavity (case 2). **(C)** Gas temperature estimates by fitting the experimental and the simulation spectra of OH [A^2^Σ^+^(*υ*′ = 0) → X^2^Π (*υ*″ = 0)] at case 2, T_gas_ ≈ T_rot_ = 310 K. APPJ, atmospheric-pressure plasma jet; OES, optical emission spectroscopy.

An example of the plasma treatment process is shown on the left in Figure 2A. There was no electric shock and burning feelings when human fingers were continuously in contact with the plasma plume used for the treatment. To identify the composition of the plasma, the OES of the plasma glow within 200–900 nm range were collected (Figure 2B). It can be clearly seen that RNS (N_2_^+^ and NO) and ROS (OH and O) are generated. Moreover, when the plasma jet effluent gas is sprayed directly into a rat’s nasal cavity, the intensity of almost all the emission lines increases compared to the case when the plasma jet is ejected into the ambient air. However, no new emission lines were observed. We further estimated the gas temperature of the plasma jet based on the transition spectrum of OH [A^2^Σ^+^(*υ*′ = 0) → X^2^Π (*υ*″ = 0)] form case 2 in Figure 2C. The best fit between the experimental and simulation spectra by using LIFBASE Spectroscopy Tool (SRI International) indicates that the gas temperature is about 310 K (Figure 2C).

### Intermittent Gas Plasma Inhalation Improved Behavioral Scoring

**FIGURE 3:**
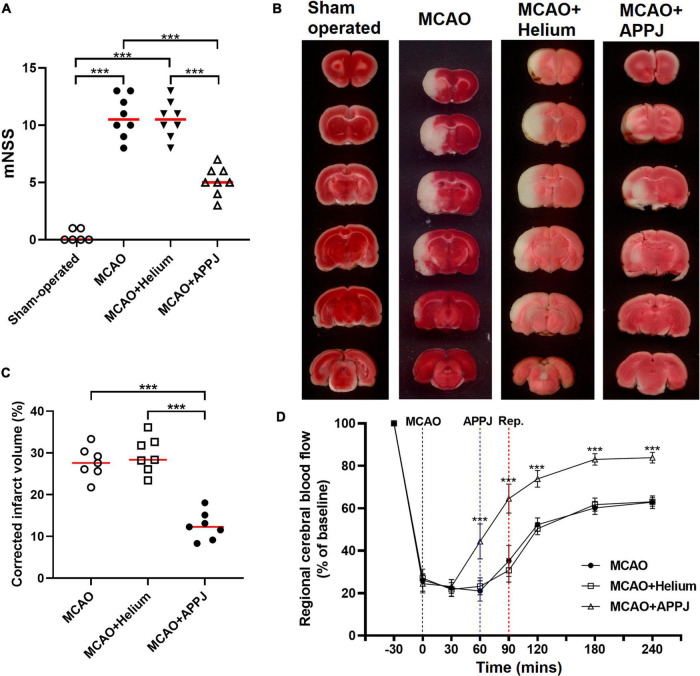
The therapeutic efficacy of the plasma inhalation on neurological severity scores, brain ischemic damage, and CBF after MCAO. **(A)**. Effect of plasma inhalation on neurological severity scores in each group. **(B)**. Representative TTC-stained sections from different groups, where the living tissues were stained red, whereas infarcted tissues were stained white. **(C)**. Quantitative analysis of TTC-defined infarct size corrected for edema, represented by the lesion volume percentage. All points stand for raw data for respective rats, whereas horizontal lines are indicative of mean. ***p < 0.001. **(D)** The CBF in the MCA region was recorded from 30 min before MCAO surgery to 4 h after the MCAO surgery. Data were represented as % of baseline value and error bars stand for SEM. ***p < 0.001 vs. the MCAO + Helium group. CBF, cerebral blood flow; MCA, middle cerebral artery; MCAO, middle cerebral artery occlusion; TTC, 2,3,5-triphenyltetrazolium chloride.

Prolonged inhalation of the plasma gas effluent will cause a significant decrease in the rat’s SpO_2_, and even lead to death of the rat due to suffocation. Therefore, rats received cycles of 5 s plasma and corresponding 5 s air inhalation to ensure the SpO_2_ values above 95% during the treatment. In addition, during the plasma inhalation, we also observed that more than 3-min intermittent inhalation could cause shortness of breath in some rats. Therefore, we selected the intermediate 2-min intermittent inhalation of plasma to minimize the impact on rats’ normal physiological indicators. The effects of APPJ treatment on MCAO rats were first evaluated according to the behavioral scoring. The plasma used in this study was generated by He discharge. Therefore, we first compared the data from MCAO and MCAO + He groups to exclude the He gas effect on MCAO rats. The difference was not significant in MCAO compared with MCAO + He groups. The rats from the sham-operated group showed very little neurological deficits at 24 h. The He-treated MCAO group displayed significant neurological deficits with the mNSS reaching 10.50 ± 0.57. Compared with the He-only-treated group, the APPJ treatment significantly ameliorated the behavioral deficit and reduced the mNSS to 5.13 ± 0.44 (*p* < 0.001, Figure 3A).

### Intermittent Gas Plasma Inhalation Increased Regional Cerebral Blood Flow

The regional CBF of the rats in each group was recorded. As shown in Figure 3D, the CBF was decreased to about 26% of the baseline value after the MCAO surgery. APPJ intermittent inhalation significantly increased the relative CBF value to 44.38 ± 8.23%, whereas He inhalation has no effect on the CBF. After reperfusion, the relative CBF value was significantly elevated and gradually stabilized, and relative CBF value in the MCAO + APPJ group (83.04 ± 2.69%) was higher than that in the MCAO (60.15 ± 3.05) and MCAO + He (61.73 ± 3.09%) group.

### Intermittent Gas Plasma Inhalation Decreased Brain Infarction After Reperfusion

Brain infarction was evaluated using neuropathological analysis after 24 h reperfusion. The difference of the MCAO group was not significant compared with the MCAO + He group. TTC staining demonstrated that intermittent inhalation of APPJ reduced the eventual volume of rat brain infarction resulting from the experimental ischemic stroke. At 24-h post-MCAO (90 min), the volume of infarction determined by TTC staining was 29.51 ± 1.62% and 12.52 ± 1.27% in He and APPJ groups, respectively (*p* < 0.001, Figures 3B,C).

### Intermittent Gas Plasma Inhalation Ameliorated Ischemic Stroke Progression

**FIGURE 4:**
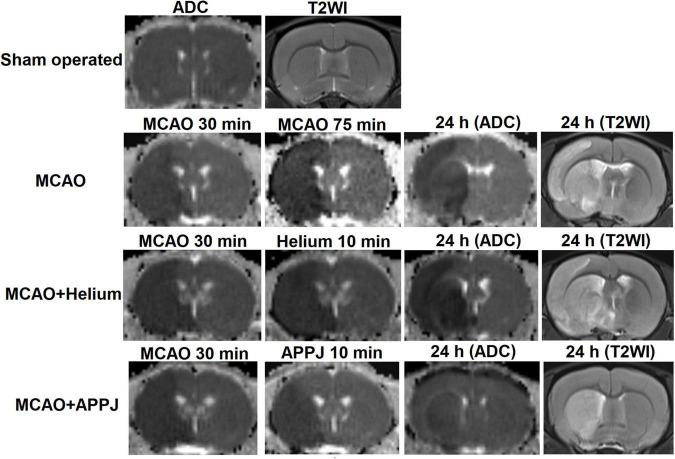
Representative MRI images of different groups at different time points.

To explore the reason for the treatment effect of APPJ intermittent inhalation on MCAO rats, we monitored the brain ischemia progression during MCAO using MRI. As shown in Figure 4, there was no abnormal signal in ADC and T2WI images in the sham-operated group during this study. Ischemic lesion zone within MCAO territory was determined by ADC lesions.

**FIGURE 5:**
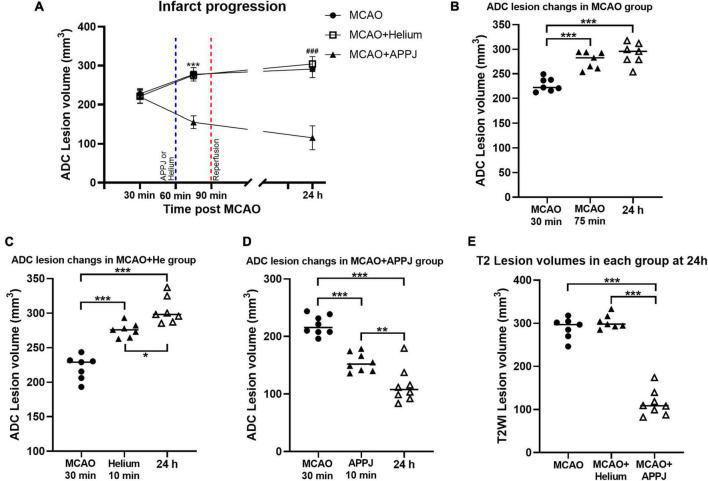
Quantitative analysis of the MRI results. **(A)** Temporal evolution of ADC-defined ischemic lesion volumes in each group. The difference of MCAO group was not significant compared with MCAO + Helium group at every time point. Values were the mean ± SEM. ***p < 0.001 compared with MCAO + APPJ group after APPJ inhalation. ^###^*p* < 0.001 compared with MCAO + APPJ group at 24 h. **(B–D)** ADC-defined ischemic lesion volume changes at different time points in MCAO, MCAO + Helium, and MCAO + APPJ groups. The points stand for raw data for all respective rats, whereas the horizontal line indicates the mean value of each group. *p < 0.05, **p < 0.01, and ***p < 0.001. **(E)** Volume of T2WI lesion for all groups at 24-h post-MCAO. The points stand for raw data for all respective rats, whereas horizontal line is indicative of mean. ***p < 0.001. APPJ, atmospheric-pressure plasma jet; ADC, apparent diffusion coefficient; MCAO, middle cerebral artery occlusion.

At 30 min after the MCAO, ADC lesions could be observed mainly at the striatum and frontoparietal cortex in MCAO, MCAO + He, and APPJ treatment groups, and differences among three groups were not significant, which were 227.70 ± 5.09, 221.30 ± 6.54, and 219.6 ± 5.99 mm^3^, respectively (*p* > 0.05, Figure 5A). Differences in values between MCAO and MCAO + He groups were not significant at every time point. The ADC-defined ischemic lesions were partially recovered after the APPJ treatment compared with the He-treated group, which were 275.6 ± 3.941 and 155.10 ± 5.78 mm^3^, respectively (Figure 5A, *p* < 0.001). No changes were observed in T2WI in each group within 90-min MCAO (results not shown). At 24-h post-MCAO, ADC lesion was enlarged, with T2WI lesion in MCAO and MCAO + He groups, whereas the APPJ treatment significantly reduced ADC and T2WI lesions. The final ADC and T2WI lesions at 24 h were 290.90 ± 8.32 and 288.90 ± 9.11 mm^3^ in the MCAO group, 304.00 ± 7.39 and 303.60 ± 6.21 mm^3^ in the He-treated group, and 115.30 ± 10.85 and 114.70 ± 10.60 mm^3^ in the APPJ treatment group, respectively (*p* < 0.001, Figures 5B–E). Figures 5B–D clearly shows the ischemic volume progression in the MCAO and MCAO + He groups, whereas the APPJ treatment significantly decelerated the ischemic volume increasing and reduced the final lesion volume at 24 h (*p* < 0.001).

### Intermittent Gas Plasma Inhalation Improved Morphology of Brain Tissues From Middle Cerebral Artery Occlusion Rats

**FIGURE 6:**
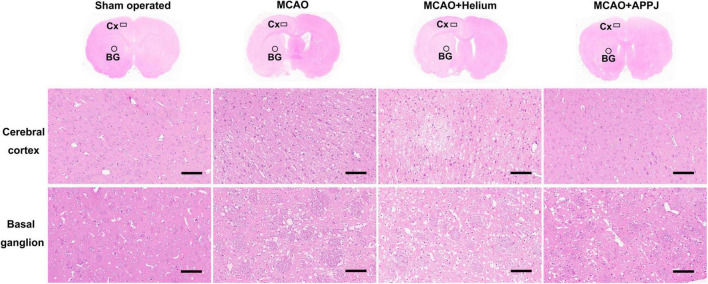
Tissue damage in each group was examined using the HE staining at 24-h post-MCAO. The representative overview and × 200 HE staining images of each group were provided. The magnified view for the cerebral cortex (Cx) and basal ganglion (BG) regions was shown as indicated by the boxes and circles, respectively, in each group. Results showed that the gas plasma inhalation improved MCAO-induced pathological changes. Scale bars show 100 μm. HE, hematoxylin and eosin; MCAO, middle cerebral artery occlusion.

The HE staining was performed to observe the morphology and pathological changes of the brain tissues in each group. The representative HE staining images of each group are shown in Figure 6. The magnified view for the cerebral cortex (Cx) and basal ganglion (BG) regions was selected near the boundary of the lesion regions based on MRI results and was indicated by the boxes and circles, respectively, in each group. There were no apparent pathological changes in the sham-operated group, cells were arranged regularly, and normal capillary morphogenesis could be observed. The structures of neurons were clear with round, large, and regular nuclei. In the Cx and BG regions of MCAO and MCAO + He groups, most cells were arranged disorderly, whereas the loose tissue structure and the pyknotic nuclei could be observed, which could be attenuated by the APPJ inhalation. The pathological changes of the lesion regions in each group were consistent with ADC and T2WI results as shown in Figure 4.

## Discussion

In the current study, we used MCAO to establish the experimental ischemic stroke model in rats and found that 90-min MCAO would lead to significant brain infarction after 24-h reperfusion. Then, we found, for the first time, that intermittent gas plasma inhalation during MCAO improved behavioral scoring, ameliorated neurological function, increased CBF, and decreased brain infarction of experimental ischemic stroke rats. Further serial ADC and T2 MRI were applied and revealed that intermittent plasma inhalation during MCAO could limit the ischemic zone progression, which may contribute to the treatment efficacy of APPJ treatment on MCAO rats.

The MRI offers a non-invasive way to track the ischemic cerebral injury progression. In the current study, we monitored the brain ischemic lesions at 30 min after MCAO, 70 min after MCAO (10 min after the APPJ treatment), and 24 h after MCAO using MRI. The results showed that the APPJ treatment significantly reduced the ischemic core progression, which may benefit to decrease the final volume of infarction and improve the neurological function at 24 h. To be specific, the infarction core, which is characterized by a zone of irreversible cell death, starts to grow within minutes of the onset of the stroke. The brain tissues will be rapidly damaged from the ischemic core to the surrounding tissues induced by the interruption of the blood flow. The neuroprotective agents could protect the tissues threatened by ischemia and limit the ischemic zone progression, which could save more time for the reperfusion therapies and improve the final prognosis (Karsy et al., 2017; Baron, 2018). Therefore, our study suggested that early intervention through plasma inhalation could impede the ischemic core growth, which may benefit the positive outcomes of ischemic stroke patients.

For the first time, we applied the APPJ intermittent inhalation on rats *via* the nasal cavity for the CNS disease treatment. A previous study (Chihiro et al., 2014) has shown the therapeutic effect of plasma inhalation on cardiovascular disease. In this study, plasmas in the O_2_ and air-gas mixture were used for inhalation by rat models of myocardial infarction (MI) through a mechanical respirator. It was revealed that the plasma inhalation increased the NO concentration in the abdominal aorta and SpO_2_ level in the blood, which may contribute to the beneficial effect in restoring heart function following MI (Chihiro et al., 2014). In the current study, we made rats to inhale gas plasmas in a more direct way and kept the SpO_2_ level more than 95% during the treatment process. Our study demonstrated that plasma inhalation could reduce brain infarction in a rat ischemic stroke model.

Among the many active species generated by plasma, NO-related species are very effective and relevant to both physiological and pathophysiological functions of CNS. The atmospheric-He plasma emissions also had NO. The strong local electric field in the jet head ionizes the air diffused into the He flow during the plasma propagation process. In the current study, we use the OES to detect the components generated in the plasma jet qualitatively. N_2_^+^ and NO could be found in the OES. Furthermore, N_2_^+^ particles could be finally converted to NO by a serious reaction with the O_2_ and N_2_ in the air (He et al., 2021). Previous studies from us and other groups have also shown that APP treatment could lead to upregulation of intracellular and extracellular NO productions (Yan et al., 2012; Preissing et al., 2020).

It has been confirmed that the occurrence and development of neurodegenerative diseases are accompanied by an abnormal increase in the NO content (Dawson and Dawson, 2018). On the other hand, when the NO content is at a low physiological concentration, it can act as a vasodilator factor to expand blood vessels and increase blood flow (Dormanns et al., 2016). NO is also an important signaling molecule in cells, which acts as a “second messenger” to activate intracellular signaling pathways and protect injured neuronal cells (Contestabile and Ciani, 2004). Furthermore, studies have shown that NO could physiologically maintain the blood flow of the brain under ischemic conditions (Terpolilli et al., 2012b). Numerous pharmacodynamics studies have confirmed that exogenous NO (including NO donor drugs and inhaled NO) can improve the blood flow of the surrounding tissues of the cerebral ischemic zone, thereby reducing the neuronal damage caused by cerebral ischemia, while reducing secondary cerebral edema and improving the neurological function score (Terpolilli et al., 2012a; Khan et al., 2019). Our previous study has also demonstrated the neuroprotective effect of APPJ treatment on neuronal cells damaged by OGD, a major pathological process during ischemic stroke, which was regulated by the NO production induced by the APPJ exposure and the inhibition of the apoptotic pathway. In the current study, for the first time, we applied the neuroprotective effects of APPJ on an *in vivo* ischemic stroke model and revealed the therapeutic effect on the ischemic stroke progression, neurological function, and reduction of infarction lesion volume.

It should be noted that during the reperfusion stage, the restoration of blood flow would lead to excess production of ROS to the ischemic brain, and the “reperfusion injury” was thought to play a dominant role for the brain injuries at this stage (Granger and Kvietys, 2015). Therefore, the plasma treatment at 90 min or after reperfusion could bring additional RONS to aggravate the damage to the ischemic brain. Our preliminary experiments on small samples of rats have tested the effect of the plasma inhalation at 90 min (before reperfusion) and 60 min after reperfusion, but the effect was even worse and the rat survival rate was at 24 h was significantly affected, and it needed further validation. We also hope to find a way of long-term application of plasma for rehabilitation after reperfusion and our further study will more focus on that.

Our previous studies also found that plasma treatment *in vitro* could increase the intracellular and extracellular NO production, and the *in vitro* neuroprotective properties of plasma were regulated by activating the NO/cGMP/PKG pathway and inhibiting the mitochondrial apoptosis pathway (Yan et al., 2020, 2022). According to the *in vitro* results, plasma inhalation by MCAO rats could increase residual CBF, ameliorate ischemic stroke progression, and reduce the final infarct volume, which could be related to the NO production induced by plasma. But some detailed mechanisms *in vivo* and the roles of other reactive species of plasma in ischemic injuries, and the intracellular targets, need further studies. Meanwhile, experimental conditions for producing stroke are different, our current study provides one possible way for plasma application on MCAO rats. Applications of plasmas in other stroke conditions, such as permanent MCAO model, are still in progress.

The plasma used in the current study was also generated in a He discharge, and some He gas also gone through the plasma jet. Previous studies have shown the neuroprotective effect of inert gas, especially He (Berganza and Zhang, 2013). Therefore, we set up the MCAO + He group to check whether the therapeutic effect on MCAO rats was induced by He-only inhalation. Our results showed that He-only inhalation by MCAO rats did not significantly improve the neurological function and the ischemic lesions compared with the MCAO group. These findings ensure that the therapeutic effects on MCAO rats were directly related to the effects of the plasma inhalation. Previous studies have reported that He mixed with air or O_2_ and inhaled by rats in ischemic stroke model during or post-MCAO for minutes or hours, which could produce neuroprotective effect and improve neurologic outcome in MCAO rats (Pan et al., 2011; Haelewyn et al., 2016). The way and duration for rats’ inhalation were different in our current study. Our study demonstrated that only 2 min of intermittent gas plasma inhalation, rather than He-only, could limit the ischemic lesion progression, improve the neurological function, and decrease brain infarction in MCAO rats.

Although our current study first demonstrated the application of intermittent inhalation of the gas plasma on an ischemic stroke rat model, more work is still needed for future studies, e.g., to track the quantified details of the active species of the plasma, explain how the reactive species enter the circulatory system and act on the brain tissues impaired by MCAO, and reveal the effective *in vivo* targets and mechanisms of the plasma on MCAO rats. Meanwhile, our study only evaluated the effect of plasma inhalation on MCAO rats at 24 h after reperfusion. However, reperfusion injury and the other pathologic cascades (energy failure, ionic imbalance, excitotoxicity, calcium overload, cytotoxic and vasogenic edema, and so on), evolving from hours to weeks, and also the glial scar is completed between 2 and 4 weeks after stroke (Brouns and De Deyn, 2009; MartInez-Coria et al., 2021), which need our further studies to clarify the long-term treatment effects of plasma. Furthermore, it would be important and interesting to explore whether treatment at other time points or even in a permanent MCAO model, which deserves our further studies. Our current study provided important insights into the timing and specific protocol of the plasma application in the rat model with experimental ischemic stroke. These findings contribute new insights into the potential medical applications of APP in combination with therapeutic treatments of ischemic stroke and other neuronal diseases.

## Conclusion

Despite decades of research, effective neuroprotection therapies remain the holy grail of ischemic stroke and other neuronal disease treatments (Schmidt-Pogoda et al., 2020). The ability to protect the ischemic brain from injuries due to the interruption of blood flow until the subsequent reperfusion treatment and then to protect the brain from any possible secondary injuries could improve the therapeutic effects and the prognosis among patients with stroke.

Previous studies have verified the neuroprotective effect of APPJ treatment *in vitro* (Mitra et al., 2020; Yan et al., 2020; Tian et al., 2021). However, whether the neuroprotective properties of plasmas were beneficial for the ischemic stroke treatment, and how to use the plasmas in animal models of ischemic stroke have not been clearly demonstrated. Our current study for the first time demonstrated that only 2 min of intermittent inhalation of the gas plasma generated by needle-aluminum ring He plasma jet discharge could limit the ischemic lesion progression, improve the neurological function, and further reduce the brain infarction in rats with experimental ischemic stroke.

This study also noted that the plasma jet was applied after the onset of the brain ischemic, suggesting that the gas plasma inhalation treatment might be a complementary combination treatment method for patients with ischemic stroke in pre-hospital stroke care, but adequate studies in humans are needed because most studies in animal models frequently failed in clinical trials (DeGraba and Pettigrew, 2000; Schmidt-Pogoda et al., 2020).

## Data Availability Statement

The raw data supporting the conclusions of this article will be made available by the authors, without undue reservation.

## Ethics Statement

Each animal experiment was approved by the Animal Care and Use Committee at Beijing Neurosurgical Institute (No. 201902030).

## Author Contributions

XY, FY, and JO conceptualized the study and contributed to resources. YC, BY, and LX contributed to methodology. ZS contributed to software, formal analysis, and visualization. BY and RH contributed to validation. YC and BY investigated the study. RH contributed to data curation. XY, BY, and YC contributed to writing the original draft. KO contributed to writing, reviewing, and editing the manuscript. JO contributed to supervision. FY contributed to project administration. XY contributed to funding acquisition. All authors have read and agreed to the published version of the manuscript.

## Conflict of Interest

The authors declare that the research was conducted in the absence of any commercial or financial relationships that could be construed as a potential conflict of interest.

## Publisher’s Note

All claims expressed in this article are solely those of the authors and do not necessarily represent those of their affiliated organizations, or those of the publisher, the editors and the reviewers. Any product that may be evaluated in this article, or claim that may be made by its manufacturer, is not guaranteed or endorsed by the publisher.

## Footnotes

1
https://www.radiantviewer.com
